# A Novel Control Method for Rotary Blood Pumps as Left Ventricular Assist Device Utilizing Aortic Valve State Detection

**DOI:** 10.1155/2019/1732160

**Published:** 2019-12-11

**Authors:** Dmitry Petukhov, Leonie Korn, Marian Walter, Dmitry Telyshev

**Affiliations:** ^1^National Research University of Electronic Technology, Zelenograd 124498, Russia; ^2^Medical Information Technology, RWTH Aachen University, Aachen 52074, Germany; ^3^Sechenov First Moscow State Medical University, Moscow 119991, Russia

## Abstract

A novel control method for rotary blood pumps is proposed relying on two different objectives: regulation of pump flow in accordance with desired value and the maintenance of partial support with an open aortic valve by the variation of pump speed. The estimation of pump flow and detection of aortic valve state was performed with mathematical models describing the first- and second generation of Sputnik rotary blood pumps. The control method was validated using a cardiovascular system model. The state of the aortic valve was detected with a mean accuracy of 91% for Sputnik 1 and 96.2% for Sputnik 2 when contractility, heart rate, and systemic vascular resistance was changed. In silico results for both pumps showed that the proposed control method can achieve the desired pump flow level and maintain the open state of the aortic valve by periodically switching between two objectives under contractility, heart rate, and systemic vascular resistance changes. The proposed method showed its potential for safe operation without adverse events and for the improvement of chances for myocardial recovery.

## 1. Introduction

Implantable rotary blood pumps (RBPs) used as ventricular assist devices (VADs) can be successfully applied under heart failure conditions for circulatory support of a weakened heart in the cardiovascular system [1–4]. Despite several decades of development and significant technological improvements, RBPs are still associated with life-threatening adverse events such as pump thrombosis or nonsurgical bleeding [5–7]. A possible reason for the appearance of these events may be due to constant speed operation [8]. Along with the overall worse sensitivity to pressure changes, this operation mode is insensitive to physiological condition changes and provides a pump flow regardless of the cardiovascular system state. Thus, it leads to a number of nonphysiological and critical conditions such as under- or overpumping, resulting in back flow or ventricular collapse. On the long run, it may result in complications such as right ventricular failure, ventricular arrhythmias, thrombus formation, cardiac tamponade or pulmonary edema [9–12].

The physiological control of RBPs is considered as the most effective way to overcome previously mentioned issues. It is believed that physiological control can reduce the number of adverse events and thus improve VAD therapy.

However, there are still no appropriate physiological feedback controllers in clinical practice [13].

To date there are various control algorithms published in literature. Generally, the control is done with the regulation of pump flow according to the Frank—Starling mechanism [14–16]. Previous studies have proven the efficiency of such controllers against constant speed operation [16].

It is believed that physiological control should detect and prevent adverse events. Arndt et al. for instance use a pulsatility index of pressure difference across the pump for selecting two operation modes: full assistance and partial assistance of the ventricle. The proposed control algorithm is preload-sensitive with a suction detection mechanism [17]. A similar approach using the pulsatility ratio of pump flow and pressure head as a control index was proposed by Choi et al. [18]. Wang et al. propose a universal physiological control algorithm for axial and centrifugal VADs with suction prevention using a user-defined threshold [19]. Wu et al. proposed an adaptive control algorithm on the basis of pump current, pump pressure head and pump speed and showed its reliability for two different pumps [20]. All of these control strategies were evaluated solely in silico.

There are many algorithms aimed only at the detection of pumping states. For example, there are a plenty of algorithms aimed on the detection of aortic valve state from changes in pump flow or pump speed waveforms [21–23] because an open state of the aortic valve is considered as most preferable during ventricular support.

Considering the importance and necessity of detecting and maintaining an open state of the aortic valve some companies have incorporated controllers in their devices for the control of aortic valve opening based on intermittent decreasing pump speed to a predefined set point [17, 24]. However, these algorithms do not know the current state of the valve during speed changes.

In the current study, we present a novel control method that uses the pressure head on the pump to achieve the following objectives: (1) to achieve desired pump flow level (2) to ensure aortic valve opening which allows for safe operation of the control method such that no back flow or suction states occur. The novelty of the control method lies in the maintaining of desired pump flow with the controllable aortic valve opening unlike the algorithms used in clinical practice. The control method was evaluated in a cardiovascular system model with changes of contractility, heart rate, and systemic vascular resistance. In the following sections the in silico performance of the proposed control method and its potential in the clinical environment are presented and discussed.

## 2. Materials and Methods

### 2.1. Overview of Control Method

**Figure 1:**
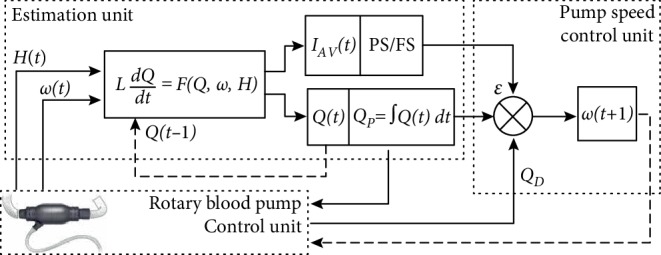
Flow chart of the control method composed of three main parts: estimation unit, speed control unit, and RBP control unit; *Q*_*P*_—pump flow per minute, *Q*_*D*_—desired pump flow, *ω*_p_—pump speed, *I*_*AV*_—index for detection of partial support (PS), and full support (FS) pumping states.

In Figure 1 a flow chart of the control method is depicted. It is divided into three main parts. The first one is the estimation unit which involves the mathematical model of the RBP. The mathematical model utilizes the pressure head (*H*) and the pump speed (*ω*) as input parameters for the estimation of pump flow waveform *Q*(*t*). It is also used for the detection of pumping states like partial support (PS) or full support (FS) with specific indices like *I*_*AV*_ .

The indices are calculated from the waveforms of derivatives obtained from the mathematical model and represent the processed (i.e., minimum or maximum or combined) value of the derivatives at the last heartbeat within a time period of five heart cycles. The indices are further used for the detection of pumping states like full support with aortic valve nonopening and for the decision about pump speed change.

The estimated pump flow *Q*(*t*) is integrated over five heart cycles, then approximated to liters per minute (*Q*_*P*_) and compared to the desired flow level *Q*_*D*_ in the pump speed control unit after five heart cycles. Therefore, in the second part of the control method, the new speed value is set, depending on the difference between estimated and desired pump flow: if the estimated flow is less than the desired, the pump speed is increased. If the estimated flow is equal or higher than the desired, the pump speed is determined by the pumping state control. In turn, the pumping state control should ensure partial support by reducing pump speed if the pump flow results in full support preventing opening of the aortic valve. So these two controllers are in conflict. It is worth to mention that during partial support pump flow may be too low, i.e., about 1–2 L/min. In order to overcome these issues we propose the following scheme.

The flow controller increases pump speed in steps of 200 rpm every five heart cycles in order to achieve the desired flow level. During this operation, the pumping state generally changes to full support. At the transition between partial and full support the speed value of the pump is stored. The pump operates in this mode for ten heart cycles. Then the speed drops 10% below the stored speed value in order to maintain partial support—as the pump operates in this mode for another ten heart cycles. So the pump speed is modulated between two levels for short periods of time: one level provides the desired pump flow and the second level ensures partial support. Thus, the system generates semi-physiological flow by providing the desired flow level and maintaining partial support.

The third part in Figure 1 shows the rotary blood pump control unit which sets a new speed value and feeds the new pressure head and speed values into the estimation unit.

### 2.2. RBPs Mathematical Models

The mathematical models for two generations of the Sputnik RBP [25] are presented here. Both pumps are a Russian design intended for use as a bridge to transplantation; the first generation of pumps is in clinical practice now, the second generation with improved weight-size parameters and energy consumption is currently going through in vivo trials. Based on the previously developed algorithm for system identification [26], the following improved mathematical models were obtained. The initial equation was set from the known relationship between pump flow, speed, and pressure head [27] as follows:

(1) $$\begin{array}{c} L\frac{dQ}{dt}=aQ+b\omega^{2}+cH, \end{array}$$

where *L* is a parameter characterizing the fluid inertia effect on the pump, *Q* is pump flow, *ω* is pump impeller speed, *H* is the pressure head across the pump and *a*, *b*, and *c* are equation coefficients. The initial equation was extended by sequentially adding terms of the form *kω*^*x*^*H*^*y*^*Q*^*z*^, where *k* is a coefficient and *x*, *y*, and *z* are integers ranging from −2 to 4. After that, all coefficients were optimized in accordance with objective function and the set of equations was obtained. From this the equations were selected satisfying the performance criteria and providing a total error of flow estimation less than 0.25 L/min in the wide range of pump speeds [26].

The final equation for Sputnik 1 is:

(2) $$\begin{array}{c} L\frac{dQ}{dt}=aQ+b\omega^{2}+cH+dQ\omega^{2}, \end{array}$$

and for Sputnik 2:

(3) $$\begin{array}{c} L\frac{dQ}{dt}=aQ+b\omega^{2}+cH+dQ\omega^{2}+eQ^{-1}\omega^{2}H+f\omega^{2}H^{2}. \end{array}$$

Table 1 with the list of coefficients is listed above.

### 2.3. Cardiovascular System Model

A mathematical model of the cardiovascular system was used for the investigation of the above described RBP models with the aim of pumping state detection. As a basis, the mathematical model from a previously published study [28] was taken. This model consists of lumped parameter models of the total circulation including the systemic, pulmonary, and peripheral parts, both ventricles with the mitral, aortic, tricuspid, and pulmonary valves. Heart rate was set to 80 bpm and RBP was connected to the left ventricle and the aorta; the speed of the pump impeller was set as a constant value.

According to Martina et al. the cardiovascular system model simulates hemodynamics of the cardiovascular system in heart failure state [28].

### 2.4. Pumping State Detection

The approach for detection of pumping state is described in more detail in [29]. This approach involves specific indices for detection. The value of every index represents the maximum, the minimum, or the combined value of the derivative during the last cardiac cycle of five heart cycles time range. All of the derivatives are obtained from the RBP mathematical model.

The idea of the approach is the following: when pump speed increases within a pumping state, the index value may increase. With further speed increase the pumping state changes and the index may behave the opposite way, for example, decrease. Thus a change in index may conform to the change of pumping states at pump speed variations. In this case, the index minimum or maximum corresponds to the speed value at the transition of pumping states such as partial and full support. The control of the aortic valve is based on the described approach.

The index *I*_*AV*_ is proposed for the detection of partial and full support. The values of the index *I*_*AV*_ for Sputnik 1 and 2 (S1 and S2) are listed in Table 2, where max and min designate the maximum and minimum values of the derivatives during a cardiac cycle. The index values were found empirically by the sequential selection of various indices on the basis of the derivatives, obtained from the RBP models.

We define the accuracy *δ*_*PS*_ of pumping state detection as follows:

(4) $$\begin{array}{c} \delta_{PS}=\left(1-\frac{\left|\omega_{t}-\omega_{i}\right|}{1000}\right)\cdot 100\text{\%}, \end{array}$$

where *ω*_*t*_  (rpm) is a target speed at which the transition between pumping state actually occurs, *ω*_*i*_  (rpm) is the speed at which the transition between states occurs according to index and 1000 is the normalizing coefficient which defines speed amplitude.

### 2.5. Simulation Routine

The performed simulations were split into three main parts: the first part consisted of contractility changes, the second one consisted of heart rate changes and the third one consisted of systemic vascular resistance changes.

**Figure 2:**
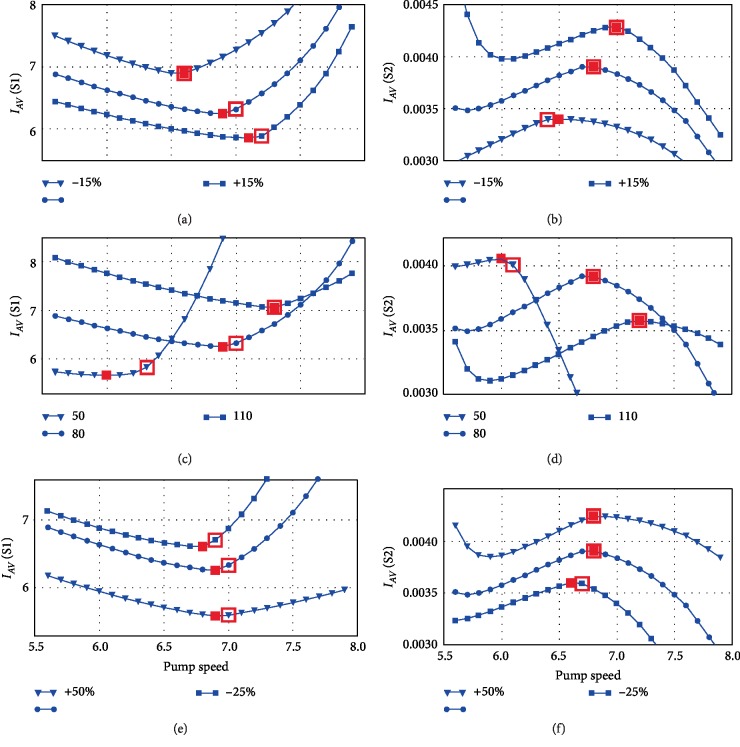
An example of partial support and full support detection for contractility (a, b), heart rate variations (c, d) and systemic vascular resistance (e, f) in the cardiovascular system for rotary blood pumps Sputnik 1 (a, c, e) and Sputnik 2 (b, d, f); *I*_*AV*_—index for the detection of aortic valve state listed in Table 2.

The following examples depicted in Figure 2 show the possibility of partial and full support detection where contractility changes in the range of ±15% (top part), heart rate changes from 50 bpm to 110 bpm (middle part) and systemic vascular resistance from +50% to −25% (bottom part) in relation to the baseline value. The range for systemic vascular resistance was chosen to avoid convergence issues at the cardiovascular system model. The small red markers in Figure 2 denote a pump speed at which the maximum or minimum value of *I*_*AV*_ index is achieved. Big unfilled red markers denote a pump speed at which the transition between partial and full support occurs—the lower speed values correspond to partial support and higher speed values correspond to full support. The position of these markers corresponds to a near-zero aortic valve flow which was determined from the integrated aortic valve flow waveform at different pump speeds. So for Sputnik 1, if the pump speed is increased and the index decreases this is equal to partial support with an open aortic valve. However, if pump speed is increased and the index increases as well this corresponds to full support (Figure 2(a)). In this case, the accuracy of pumping state detection is determined by the degree of conformity between small and big red markers according to Equation (4).

## 3. Results

A more detailed look on the accuracy of pumping state detection according to Equation (4) at various contractility, heart rates, and systemic vascular resistance in the cardiovascular system is presented in Table 3.

The mean overall accuracy for the detection of states is about 91% for Sputnik 1 and 96.2% for Sputnik 2, whereby the minimal accuracy is not less than 70%.

**Figure 3:**
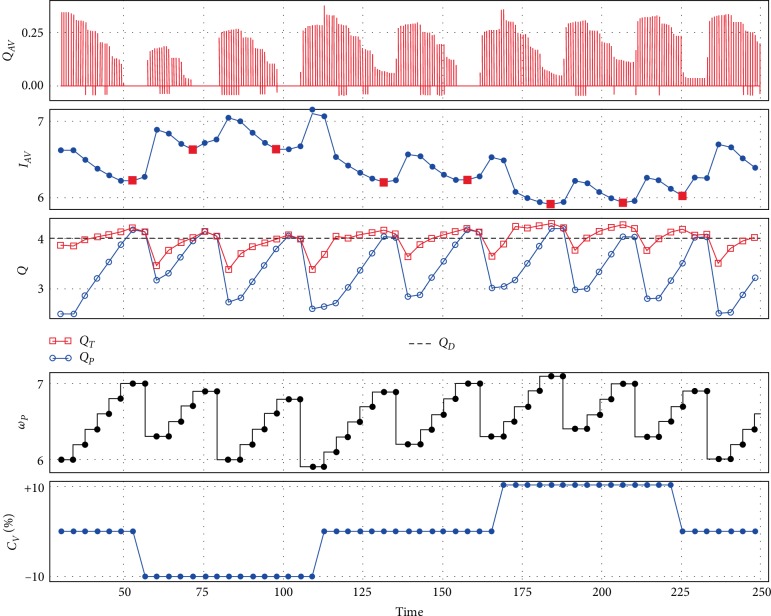
An example of control method performance with Sputnik 1 during contractility changes: control method tries to maintain approximated pump flow near 4 L/min along with maintaining the partial support state which leads to a periodic pump speed decrease in accordance with the *I*_*AV*_ index; *Q*_*AV*_—aortic valve flow (L/s), *I*_*AV*_—index, *Q*_*T*_—total flow (L/min), *Q*_*P*_—approximated pump flow (L/min), *Q*_*D*_—desired pump flow (L/min), *ω*_*P*_—pump speed (rpm), *C*_*V*_—contractility (%).

Figure 3 shows the performance of the control method with Sputnik 1 during contractility variations. The flow through the aortic valve, pump speed, pump flow, and the total flow are depicted.

To achieve the desired pump flow *Q*_*D*_, the speed is continuously increased by 200 rpm every five heartbeats. However, this may lead to a full support. The transition to full support is determined as an increase of the index *I*_*AV*_ during pump speed increase. In other words, the transition from PS to FS corresponds to a local minimum of the index *I*_*AV*_ during the increase in pump speed. These minimums are denoted by the red square markers in the *I*_*AV*_ diagram.

When the desired flow is achieved then the control method may decrease speed to ensure PS if the current pumping state is FS. In this case pump speed decreases by 10% relative to the speed at the transition to FS designated by red marker, i.e., up to 5800–6000 rpm. As a consequence, the transition to partial support with an open aortic valve takes place. This can be observed in Figure 3 in the *Q*_*AV*_, and *I*_*AV*_ diagram.

This is also an example of prioritization between two distinct controllers: it is desirable to achieve desired pump flow, although the aortic valve should remain open. The control method provides two types of control for short periods of time.

The contractility changes cause a variation in the absolute value of the index *I*_*AV*_ but partial and full support states are still detected by finding of the minimum of the index at pump speed changes.

**Figure 4:**
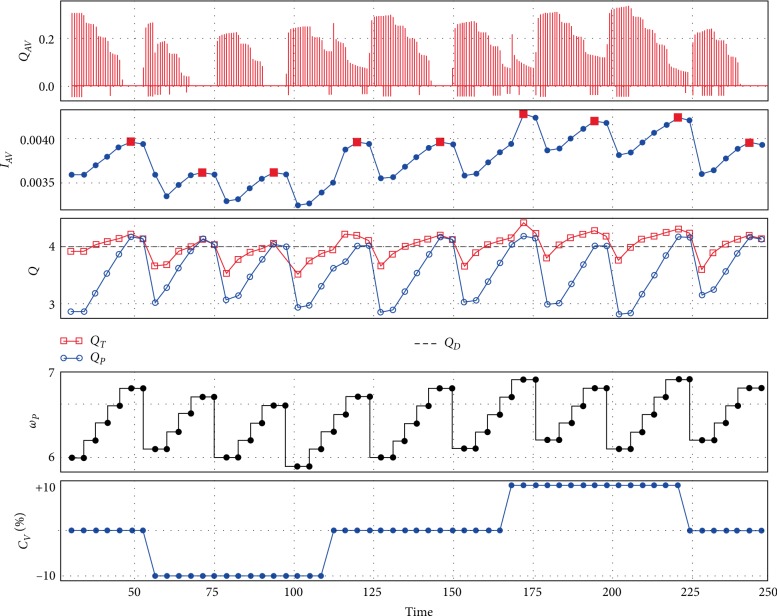
An example of control method performance with Sputnik 2 during contractility changes: control method tries to maintain approximated pump flow near 4 L/min along with maintaining the partial support state which leads to a periodic pump speed decrease in accordance with the *I*_*AV*_ index; *Q*_*AV*_—aortic valve flow (L/s), *I*_*AV*_—index, *Q*_*T*_—total flow (L/min), *Q*_*P*_—approximated pump flow (L/min), *Q*_*D*_—desired pump flow (L/min), *ω*_*P*_—pump speed (rpm), *C*_*V*_—contractility (%).

Figure 4 shows the performance of control method during contractility variations for the pump Sputnik 2. It is similar to Figure 3 with the exception that a transition to full support is determined from the maximum of the index *I*_*AV*_. A contractility increase also causes an increase in total flow.

**Figure 5:**
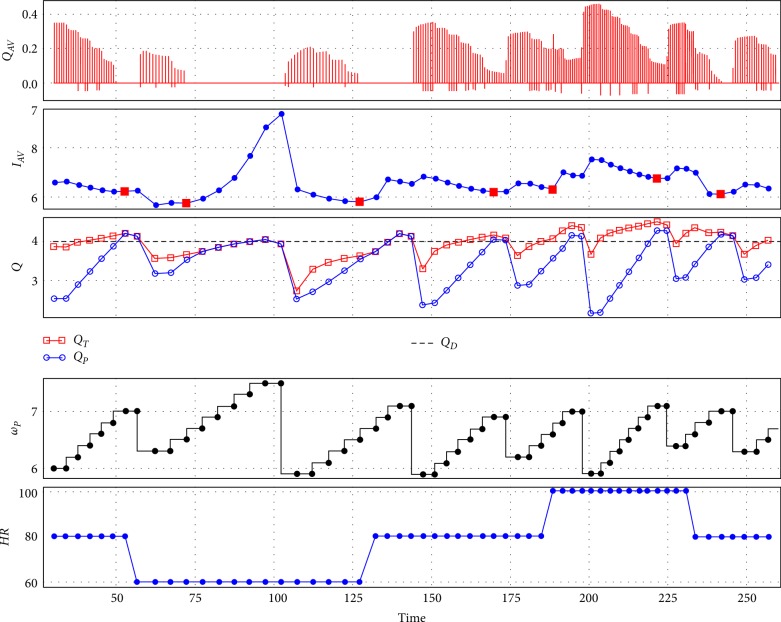
An example of control method performance with Sputnik 1 during heart rate changes: control method tries to maintain approximated pump flow near 4 L/min along with maintaining the partial support state which leads to a periodic pump speed decrease in accordance with the I_AV_ index; *Q*_*AV*_—aortic valve flow (L/s), *I*_*AV*_—index, *Q*_*T*_—total flow (L/min), *Q*_*P*_—approximated pump flow (L/min), *Q*_*D*_—desired pump flow (L/min), *ω*_*P*_—pump speed HR—heart rate (bpm).

In Figure 5 the influence of heart rate changes in the cardiovascular system with Sputnik 1 on the control are presented. In this case, the full support state is considered as an increase of *I*_*AV*_ index during an increase in pump speed. Thus, the minimum of the index corresponds to the transition from PS to FS.

With an increasing heart rate up to 100 bpm the desired pump flow level of 4 L/min is easily achieved with an open aortic valve.

**Figure 6:**
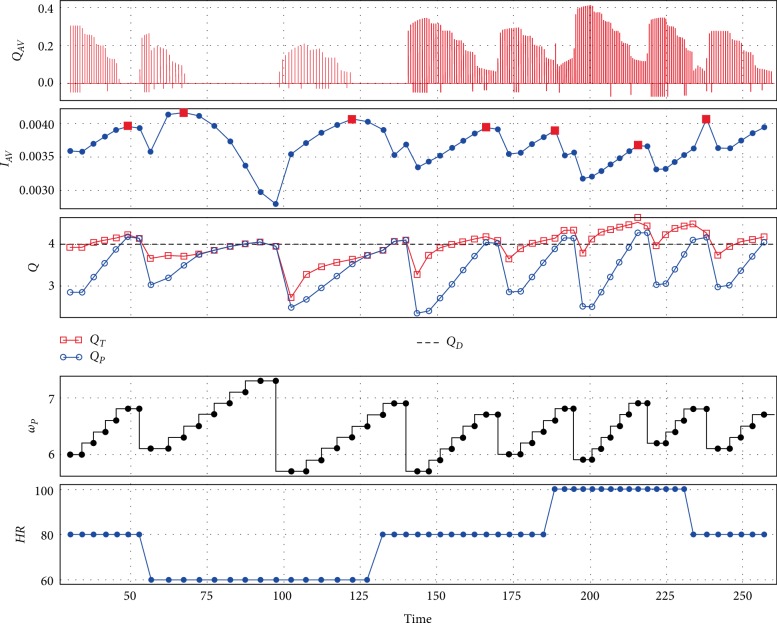
An example of control method performance with Sputnik 2 during heart rate changes: control method tries to maintain approximated pump flow near 4 L/min along with maintaining the partial support state which leads to a periodic pump speed decrease in accordance with the I_AV_ index; *Q*_*AV*_—aortic valve flow (L/s), *I*_*AV*_—index, *Q*_*T*_—total flow (L/min), *Q*_*P*_—approximated pump flow (L/min), *Q*_*D*_—desired pump flow (L/min), *ω*_*P*_—pump speed (rpm), HR—heart rate (bpm).

Figure 6 shows the performance of the control method during heart rate changes for Sputnik 2. It is similar to Figure 5 except that a transition to full support is determined from the maximum of the index *I*_*AV*_. Both pumps unload the ventricle almost in the same way, what can be seen from aortic valve flow and flow diagram *Q*.

**Figure 7:**
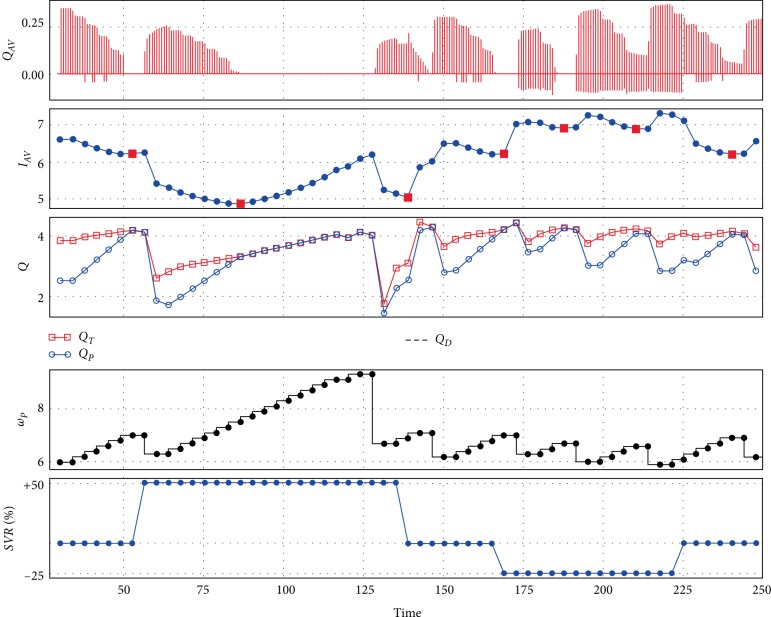
An example of control method performance with Sputnik 1 during systemic vascular resistance changes: control method tries to maintain approximated pump flow near 4 L/min along with maintaining the partial support state which leads to a periodic pump speed decrease in accordance with the *I*_*AV*_ index; *Q*_*AV*_—aortic valve flow (L/s), *I*_*AV*_—index, *Q*_*T*_—total flow (L/min), *Q*_*P*_—approximated pump flow (L/min), *Q*_*D*_—desired pump flow (L/min), *ω*_*P*_— pump speed (rpm), SVR—systemic vascular resistance (%).

**Figure 8:**
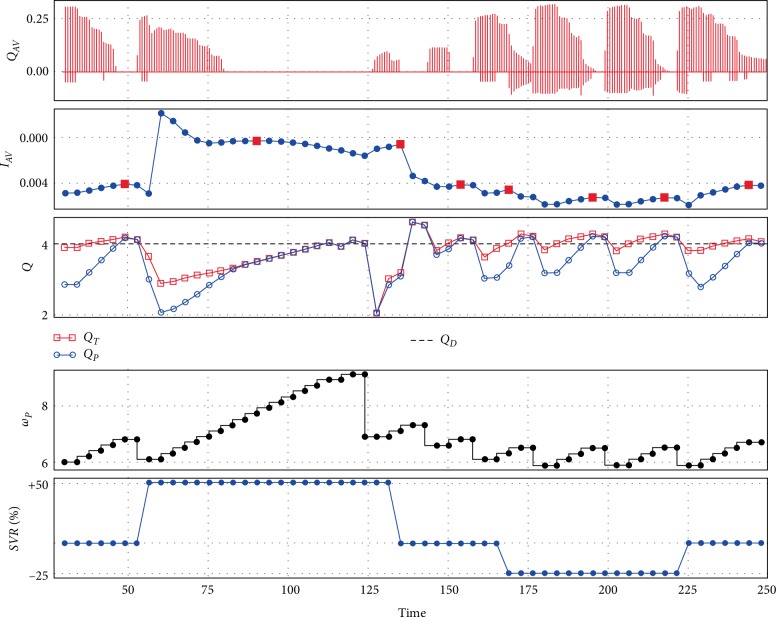
An example of control method performance with Sputnik 2 during systemic vascular resistance changes: control method tries to maintain approximated pump flow near 4 L/min along with maintaining the partial support state which leads to a periodic pump speed decrease in accordance with the *I*_*AV*_ index; *Q*_*AV*_—aortic valve flow (L/s), *I*_*AV*_—index, *Q*_*T*_—total flow (L/min), *Q*_*P*_—approximated pump flow (L/min), *Q*_*D*_—desired pump flow (L/min), SVR—systemic vascular resistance (%).

Figures 7 and 8 show the performance of the control method during systemic vascular resistance changes for the Sputnik 1 and Sputnik 2. These figures are very similar to the previous ones. It is worth to note, that an increase in resistance requires a significant increase in speed to achieve the desired flow level. At the same time, a decrease of resistance also leads to an increase in the total flow.

## 4. Discussion

The current study presents a novel control method for the detection of the aortic valve state using a mathematical model of rotary blood pumps. The method has two objectives: providing the desired pump flow level and at the same time maintaining a partial support pumping state. Thus, the control method provides semi-physiological flow and does not mimic any physiological mechanisms since these are disturbed during heart failure.

The control method has been extensively evaluated in silico. The results show that desired pump flow can be achieved and the open state of the aortic valve can be maintained in various physiological conditions by periodical changes of pump speed. The decision about pump speed change was made in a period of five heart cycles on the basis of approximated pump flow *Q*_*P*_ and the state of the aortic valve. If *Q*_*P*_ was equal or higher than the desired flow level then pumping state control was a primary objective.

Control of pumping states, in particular the aortic valve state, allows for avoidance of adverse events of RBPs in the cardiovascular system. This capability will also improve the prevention of long-term complications such as aortic valve insufficiency or thrombus formation [30, 31]. The periodic alternation between open and closed conditions will allow gradual training of heart muscle and thus reduce the risk of aortic atrophy. In the future, this can be used to establish methods for myocardial recovery [32, 33] as there is a need for advanced control strategies and consistent outpatient management [34].

Various approaches to detect the aortic valve state have been proposed in literature. For example, Ooi et al. used fourteen indices derived from the waveform of pump speed to detect an aortic valve nonopening state [23]. Hayward et al. also processed the pump speed waveform to identify the aortic valve state using data from patients with HeartWare HVAD [22]. Granegger et al. determined the aortic valve state with a specificity of 86.8% and a sensitivity of 96.5% derived from the pump flow waveform with data from animal experiments [21]. In addition, Jansen-Park et al. used data from seven sheep's to detect the moment of aortic valve closing on the basis of pump inlet pressure and pump power [35].

However, none of these approaches have been evaluated under various physiological conditions or together with pump flow regulation. In our in silico evaluation, we showed the performance of the control method with the possibility of aortic valve state detection ensures the prevention of back flow or even left ventricular suction under various conditions.

In clinical practice, an approach to detect aortic valve status was done in HeartAssist 5 [36], which uses a flow sensor to qualitatively analyze the shape of the pump flow waveform during systole. Similar approaches for the control of states with commercial RBPs are described in other studies [17].

The introduced approach for the detection of the aortic valve state is based on the assessment of changes in fluid dynamics through the pump using a mathematical model of the rotary blood pump. The approach showed reasonable accuracy of the detection according to Figure 2. It also does not require user-selectable thresholds for the detection of the states.

It is worth to note the minimal accuracy of 70% during heart rate variation for Sputnik 1. In this case, the flow through the aortic valve is underestimated, i.e., the control method estimates the transition to FS state despite having a small valve flow. This can be considered as quite acceptable and more preferable than overestimation. In the case of overestimation, a nonzero aortic valve flow is shown although it is zero. This condition indicating false partial support can be considered negative for control.

In addition, the derivatives obtained from the RBP models represent the dynamics of flow through the pump at different pump speeds. These additional signals can expand the potential of the algorithms analyzing pump signal waveforms to the detection of specific states [37, 38].

The used mathematical models of RBPs were the models of Sputnik pumps. The obtained results suggest that the presented control method can be applied to any clinical available rotary blood pump, if the mathematical model of the pump is known.

As an advantage of the proposed control method and a distinction from other methods of pumping state identification [22, 37], the possibility of indirect flow estimation using the RBP mathematical model has to be emphasized. There is need for the improvement of accuracy of pump flow estimation under dynamic conditions, as this is one of the key factors for accurately choosing pump speed [8], whereas the prospects for a direct measurement are limited.

## 5. Limitations

The authors acknowledge that the current study contains certain limitations which will be addressed in future research. For example, the pump models use the differential pressure across the pump as one of the input parameters, whereas continuous monitoring of the pressure head is not standard in real dynamic conditions after pump implantation. So far, VADs with the ability of pressure head measurement are Incor VAD and Impella [17, 39]. The referred commercial measurements of pressure head are only validated for shorter time durations, so long-term application requires further development of sensors. Therefore, there is a need to adapt the control method to the intrinsic pump parameters such as electric current, voltage, or speed. The obtained results provide a general concept which needs to be improved in the noted way. The next point is that the desired pump flow maintained for a short period of time should be adjusted to the patient demands somehow, especially during physical activity. At the current step, an increase of total cardiac output during physical activity provided by the heart only.

## 6. Conclusion

A novel control method for rotary blood pumps was proposed, which shows the possibility of using a pressure head signal to control the speed of a RBP according to different objectives: the maintenance of the desired pump flow and the maintenance of partial support with an open aortic valve. In silico results showed that the control method can periodically achieve the desired pump flow and periodically maintain the open state of the aortic valve providing a total semi-physiological flow in various physiological conditions with different rotary blood pumps. It is worth noting that maintaining open state of the aortic valve enhances aortic pulse pressure and ensures a safe operation without adverse events. Furthermore, the control method offers estimated pump flow as an important hemodynamic variable and the state of the aortic valve as additional diagnostic information about heart-pump interaction. In the next step, in vitro and in vivo experiments would be required to further evaluate and improve the control method.

## Figures and Tables

**Table 1:** List of coefficients for rotary blood pump models.

Coefficients	Sputnik 1	Sputnik 2
a	−2.0400e + 1	−1.0952e + 1
b	7.1347e − 6	5.0372e − 6
c	−3.9880e + 0	−2.6919e + 0
d	−2.1035e − 07	−2.0857e − 7
e		9.3467e − 10
f		4.8253e − 12
L	5.3037e + 0	3.6903e + 0

**Table 2:** Indices for aortic valve state detection for rotary blood pumps Sputnik; S1—Sputnik 1, S2—Sputnik 2, max—maximum value of the derivative, min—minimum value of the derivative during a cardiac cycle.

Index	S1	S2
*I*_*AV*_	max(*dQ*/*dt* · *dQ*/*dω*)/(max(*dQ*/*dt* · *dQ*/*dω*) − min(*dQ*/*dt* · *dQ*/*dω*))	max(*dQ*/*dt* · *dQ*/*dω*) − min(*dQ*/*dt* · *dQ*/*dω*)

**Table 3:** Accuracy of partial support and full support detection with the index *I*_*AV*_ according to Equation (4) at various contractility, heart rates and systemic vascular resistance in cardiovascular system; S1—Sputnik 1, S2—Sputnik 2.

Contractility, %	Accuracy, %		Heart rate, bpm	Accuracy, %		Systemic vascular resistance, %	Accuracy, %	
	S1	S2		S1	S2		S1	S2
−15	100	90	50	70	90	−25	90	90
−10	90	100	60	80	90	−15	90	100
−5	90	100	70	90	100	−10	100	90
Baseline	90	100	80 (baseline)	90	100	Baseline	90	100
5	90	90	90	90	100	15	100	100
10	80	90	100	100	90	25	100	100
15	90	100	110	100	100	50	90	100
Overall	90	95.7	Overall	88.6	95.7	Overall	94.3	97.1

## Data Availability

The original data used to support the findings of this study have been previously published: Petukhov DS, Telyshev DV. Simulation of blood flow dynamics changes through implantable axial flow pump. Biomedical Engineering. 2015;48(6):336-40. DOI: 10.1007/s10527-015-9482-1. Petukhov D, Telyshev D, Walter M, Korn L. An algorithm of system identification for implantable rotary blood pumps. Ural Symposium on Biomedical Engineering, Radioelectronics and Information Technology (USBEREIT). 2018. P. 61-63. DOI: 10.1109/USBEREIT.2018.8384550. The articles are in open-access.
